# Methane Hydrate Structure I Dissociation Process and Free Surface Analysis

**DOI:** 10.1021/acs.energyfuels.4c00267

**Published:** 2024-04-15

**Authors:** Dianalaura Cueto Duenas, Derek Dunn-Rankin, Yu-Chien Chien

**Affiliations:** † Department of Civil and Environmental Engineering, University of California, Irvine, California 92697-2175, United States; ‡ Department of Mechanical and Aerospace Engineering, University of California, Irvine, California 92697-3975, United States

## Abstract

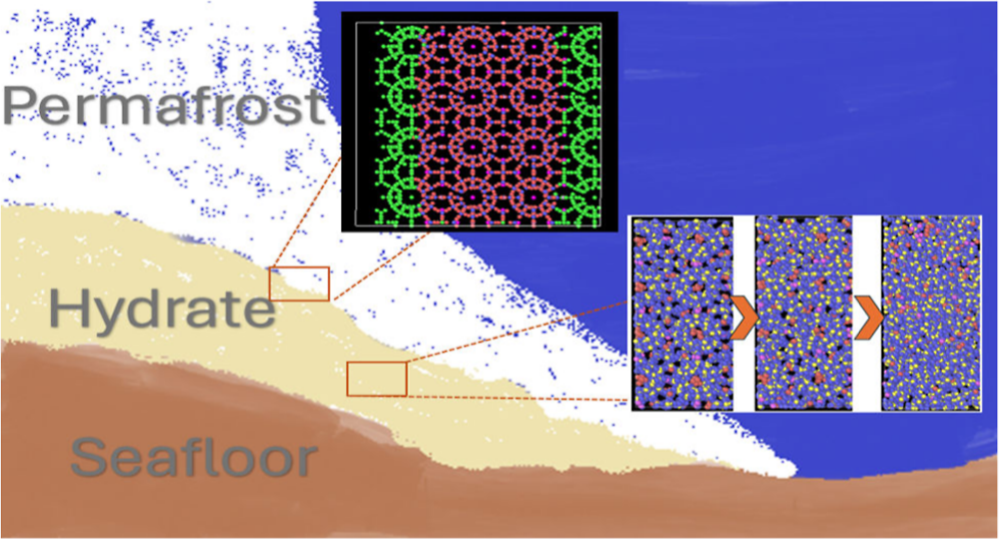

Methane hydrates are crystalline solids of water that contain methane molecules trapped inside their molecular cavities. Gas hydrates with methane as a guest molecule form structure I hydrates with two small dodecahedral cages and six tetra decahedral large cages. This study assesses the influence of occupation and the behavior of methane release from the molecular perspective during the dissociation process, particularly for the purpose of testing a series of molecular dynamics simulations. The dissociation cases conducted include an ideal 4 × 4 × 4 and 2 × 2 × 2 supercell methane hydrate system while inducing dissociation with two different types of temperature-rising functions for understanding the limitation and capability. These temperature-rising functions are temperature ramping and a single temperature step simulating in 5–7 various conditions. Temperature step results showed the earliest dissociation starting 50 ps into the simulation at an Δ*T* of 100 K, while at an Δ*T* of 80 K, dissociation was not observed. There was not a distinct dissociation preference observed between large and small cages, so it appears that the dissociation affects the entire structure uniformly when temperature increases are applied throughout the system rather than transport from a boundary. Temperature ramping simulations showed that the dissociation temperature increased with a higher heating rate. The mean-squared displacement results for the oxygen atoms in the water molecules at a high heating rate of 400 TK/s showed behavior similar to that for methane gas. As in the temperature step simulation, there were no clear differences in dissociation between large and small cages, which suggested homogeneous dissociation in all cases. Finally, a coordination analysis was performed on a 3 × 4 × 4 structure I methane hydrate with two free surfaces to demonstrate clear free surface boundaries and its location.

## Introduction

Gas hydrates with methane as a guest molecule form structure I hydrates with a unit cell containing 46 water molecules arranged on two small dodecahedral cages and six tetra decahedral large cages. An ideal structure I methane hydrate unit cell contains 8 methane molecules and one in each cage. Methane hydrates are an important potential energy source, and their occurrence is widespread across the globe, under lakes and oceans at certain temperature and pressure conditions, usually at depths above 300 m (e.g., Arctic) and under 500 m. Hydrates are also found under the permafrost where temperatures are low,^1^ and there is a wide body of literature mapping methane hydrate reserves. Global methane hydrate estimates range from tens of thousands to millions of trillion cubic feet (Tcf) of methane gas contained in hydrates. In 2011, Johnson^2^ made an assessment of every coastal margin including Polar Regions and estimated a global Technically Recoverable Resource (TRR) of 10^4^ Tcf, though no economically recoverable resources were calculated.

### Methane Hydrate Dissociation

An additional significant impact of methane hydrates is environmental. Methane hydrates are located under the permafrost in outer continental shelf areas in the deep ocean. When the temperature rises and methane gas is released from permafrost, a greenhouse gas, which is 21 times more harmful than CO_2_ per molecule, is released into the atmosphere, thus enhancing global warming. Understanding the dissociation of hydrates is an important step in avoiding negative environmental consequences of inadvertent methane release from the hydrate. In addition, and as mentioned, methane hydrates have potential as an alternative energy source with less emission because of their large quantity and widespread occurrence.^3,4^ Moreover, there are different extraction methods that have been investigated and continue to develop. Environmental impact is a major consideration of these extraction methods.^5,6^ One major and long-shot goal of methane hydrate research is to potentially extract the methane as an energy source and at the same time store CO_2_ from the atmosphere to form CO_2_ hydrate.^7^ As one step toward this goal, which is a large-scale process, it is valuable to first understand the principles of how the methane hydrate dissociation/formation process occurs. Understanding, methane hydrates from the smallest scales can be used to escalate the findings to a large scale.^8^ Prior molecular-scale methane dissociation studies have brought to light the understanding of thermodynamic properties, and they have helped understand the mechanisms as well as kinetics of methane hydrate dissociation.^9,10^ Methane hydrate dissociation is described as a two-stage process by Ding et al.^11^ The first stage of dissociation consists of the increase of diffusive behavior for the host water molecules until the lattice structure of the water cages breaks. The second stage is the escape of methane molecules from the water cages. Iwai et al.^12^ found similar results, but instead of bringing the system to a sudden temperature change, they gradually increased temperature above equilibrium at different heating rates. They also observed a two-stage dissociation process where water cages broke down first and then methane escaped. Methane hydrate cage occupancy effects were studied by Myshakin et al.^13^ They showed that increasing the ratio of empty cages decreases stability considerably, speeding dissociation and decreasing melting temperature. They found that the decomposition rate is highly dependent on the hydration number. Several methane hydrate dissociation molecular dynamics simulations were performed by Kondori et al.^14^ to study the impacts of temperature, pressure, and cage occupancy. They found that methane hydrates were less stable at higher temperatures and more stable at higher pressures. They also describe a destabilization effect with decreasing cage occupancy, as previously reported by Myshakin et al.^13^

### CO_2_/CH_4_ Exchange in Hydrate

In nature, there are two replacement mechanisms: the solid-state replacement with no hydrate dissociation and the other mechanism where CH_4_ hydrates partially or dissociates and a new CO_2_ hydrate is formed from the reformation of free water molecules. To take advantage of this spontaneous mechanism, researchers have focused on the analysis and optimization of the CO_2_ replacement mechanisms for the extraction of CH_4_, using as an energy source, with the simultaneous sequestration of CO_2_ in the deep ocean.^15,16^ Molecular dynamics simulations have been an important tool to study these phenomena. Microsecond simulations of the replacement mechanism were performed by Bai et al.,^17^ and the results showed a two-step process where the methane hydrate melts and the new carbon dioxide hydrate formation is facilitated by “the memory effect”. The role of small molecules such as nitrogen in natural environments has been explored by Matsui et al.^18^ which showed a hydrate mixture and a preferential formation in large cages compared to small cages and also a higher replacement efficiency than when pure CO_2_ was used. The previously mentioned studies^17,18^ used an interface simulation with a separate methane hydrate phase and CO_2_ phase in preparation for the interface configuration, and it is important to understand the concept of free surface and bulk behavior. Other research studies also included the use of additives such as surfactants and auxiliary gases to promote mass transfer over the hydrate film that forms in the interface.^19^ As methane hydrates are naturally found in outer continental areas, where they are exposed to liquid water and seafloor sediments, the environment can change the hydrate stability conditions. To develop efficient methane gas extraction methods, these factors are considered in this research to simulate a more realistic scenario.

The molecular simulation study presented in this work enriches insights from previous methane hydrate dissociation to replacement studies described above and, particularly, focuses on how temperature rise affects the hydrate dissociation as measured by various properties. Subsequently, the concept of free surface and hydrate bulk is studied and analyzed on hydrate structure one as a precedent to the molecular scale study of CO_2_ hydrate sequestration during the previously explained hydrate exchange process. This research work is implemented on LAMMPS^20^ and is targeted to provide perspectives on the dissociation process of methane hydrate from a molecular interaction perspective.

## Methodology

**Figure 1:**
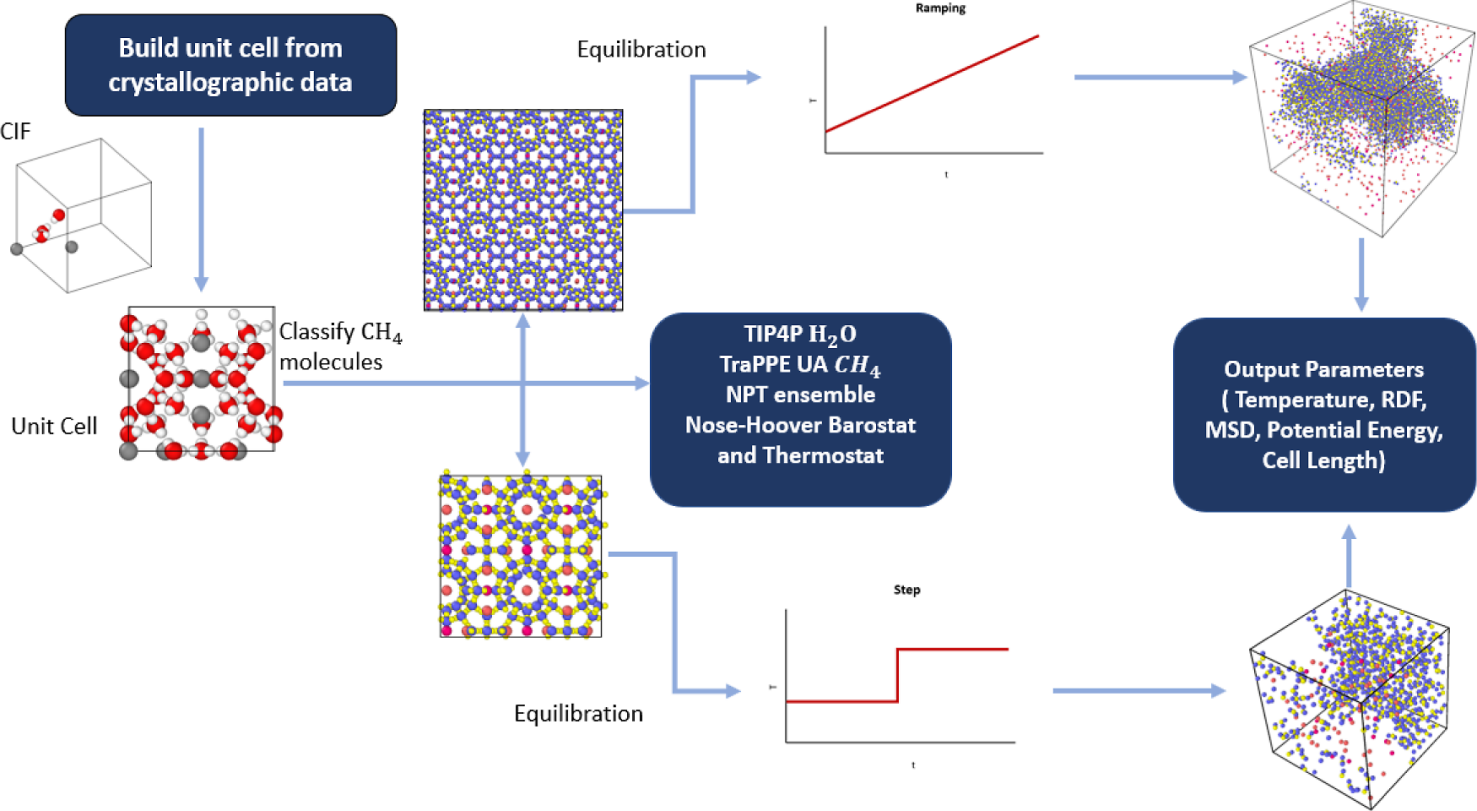
Methane hydrate dissociation molecular dynamics simulations’ methodology diagram.

The methodology of the step-by-step procedures is integrated and described in Figure 1 as a systematic flowchart for studying the dissociation process under different temperature functions. First, the simulated system is built from crystallographic data. The system is then equilibrated using appropriate force fields and undergoes temperature ramp or temperature step function for dissociation. In the final step, the parameters of interest are extracted from all simulated conditions, shown as the last part on the right in the diagram. The details of the system preparation and simulation conditions are listed in the following sections. Once the methane hydrate dissociation data are acquired and collected, it is essential to prepare hydrate as a free surface ready for the next step of dissociation and sequestration research, and the coordination analysis is needed in using an OVITO (visualization tool). Details are explained in the respective section.

### System Setup

**Figure 2:**
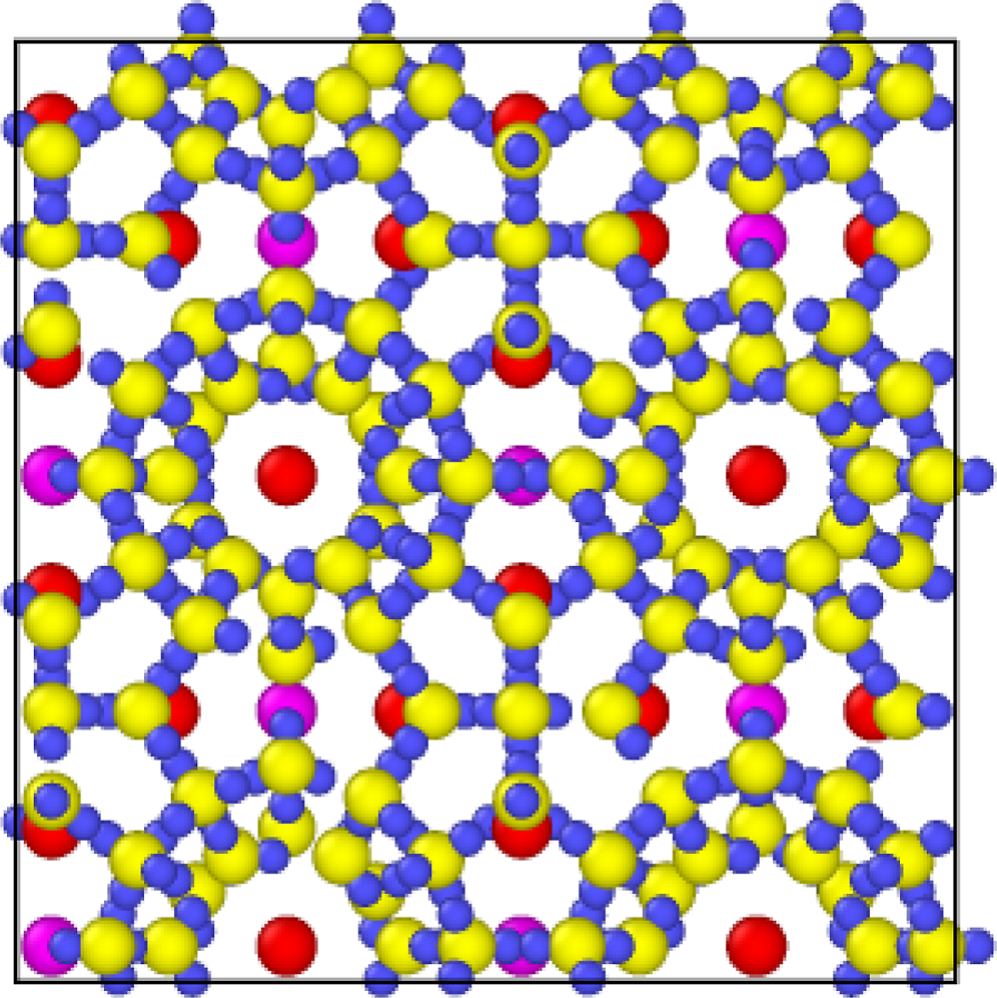
2 × 2 × 2 methane hydrate system used for temperature step simulations.

**Figure 3:**
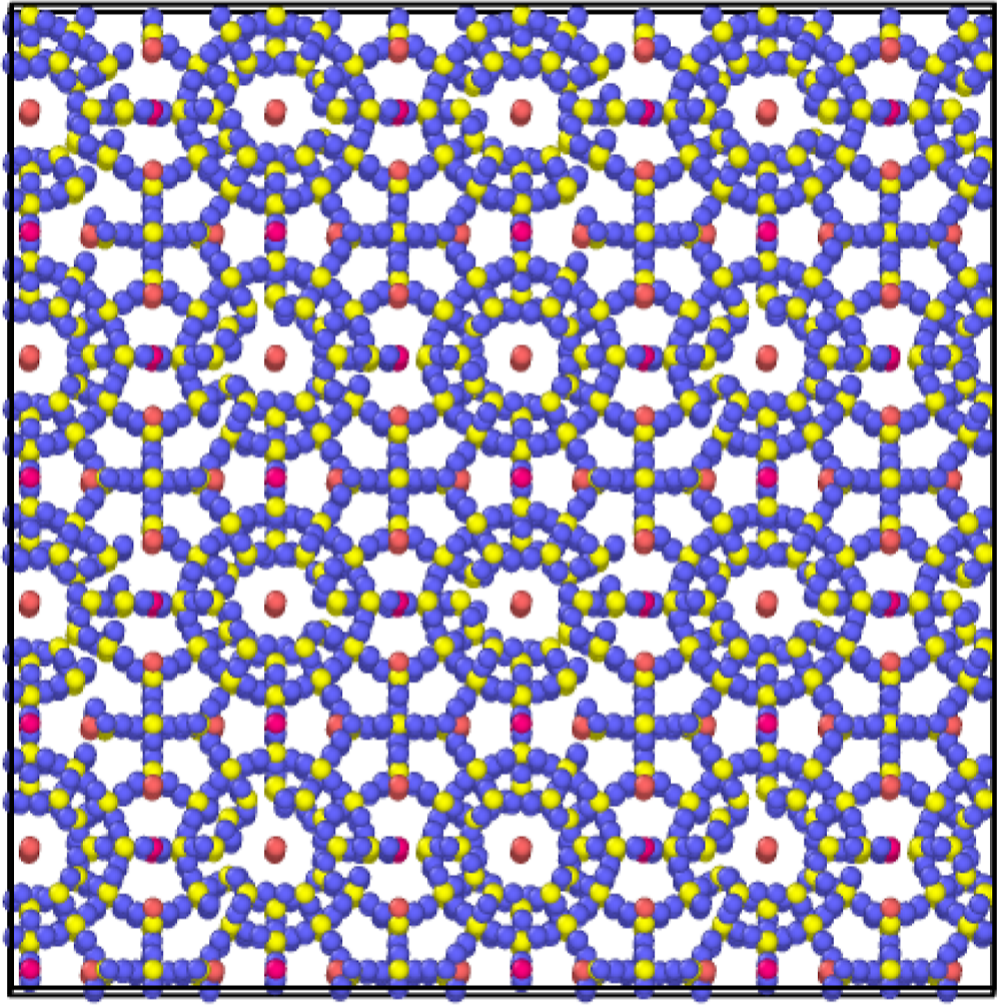
4 × 4 × 4 methane hydrate system used for temperature ramping simulations.

The initial geometry of the unit cell for methane hydrate is built using AVOGADRO,^21^ a cross-platform molecular editor. The overall steps are as follows. First, the Crystallographic Information File (CIF)^22^ is modified to eliminate the hydrogen molecules from the atomistic form of methane to obtain a coarse-grain methane form. This step can either be achieved in a Protein Data Bank (PDB) file with an appropriate file conversion header or by starting in AVOGADRO directly and then converting to a suitable format afterward. The second step is to multiply the built configuration using AVOGADRO to obtain the hydrate Unit Cell. The third and fourth step comprises exporting the PDB file and importing the data in VMD^23^ for visualization. The fifth step is to edit the PDB file to add angles, mass, and molecular data. The sixth step involves converting all of the detailed molecular information to a DATA file readable with LAMMPS. The final preliminary setup step is to multiply the unit cell in LAMMPS to create a DATA file at the desirable size. In this research, two system sizes used are 2 × 2 × 2 (Figure 2) for step simulations and 4 × 4 × 4 (Figure 3) for ramping simulations.

### Interatomic Potentials for H_2_O and CH_4_

Once the initial configuration is set up, it is important to define force fields for water and methane that describe interactions accurately for dissociation conditions. Interatomic potential models describe molecular interactions in categories such as atomistic potentials and coarse-grain models. Atomistic potentials describe the interaction of each individual atom in a molecule, while coarse-grain models describe the molecules as pseudoatoms approximating the interaction values as a group. Coarse-grain models have fewer degrees of freedom leading to shorter simulation times but also to less accuracy for some molecular properties depending on the specific model.^24^

Since the methane hydrate structure is formed by water cages and our focus is to study the structure change based on cage size, the choice of water force field can impact the simulation results. In 2006, García Fernández et al.^25^ performed molecular dynamics simulations on methane hydrate to study its melting point using 7 different water force fields, such as SPC/E, TIP4P, TIP4P-Ew, TIP4P/ice, TIP4*P*/2005, TIP5P, and TIP5P-E. They found that using the TIP4P model resulted in the lowest melting point. Jorgensen et al.^26^ studied water density by simulating a water dimer with six different water force fields, such as Bernal–Fowler, SPC, ST2, TIPS2, TIP3P, and TIP4P. The results showed that the most accurate results were obtained by the TIP3P and TIP4P force fields with 2 and 0% error, respectively. Given the accuracy of the TIP4P to represent density and a low melting point, this research uses TIP4P as the water force field model.

In order to reduce the computer resource demand, considering the low content of methane, and also because our main interest is to study water cage structure, we simplify the interaction using a coarse-grained methane model developed by the University of Michigan in 1998 called TraPPE UA.^27^ The parameters used in all of the simulations are shown in Table 1.

**Table 1:** Methane Hydrate Force-Field Parameters for Water (TIP4P) and Methane (TraPPE UA) Molecules

molecule	σ (Å)	ϵ (kcal/mol)
H_2_O	3.1536	0.1550
CH_4_	3.73	0.2941

### Simulation Conditions

Both hydrate system configurations for temperature ramping and step temperature change were equilibrated at 50 bar and 100 K, with periodic boundaries in all directions using the *NPT* ensemble, Nose–Hoover thermostat and barostat, and TIP4P and TraPPE UA to describe molecular interactions for water and methane, respectively.

A ramping temperature simulation forces a constant rate of temperature rise from a fixed initial temperature (100 K) to a fixed final temperature (500 K) over a given time. The temperature range passes through the hydrate equilibrium stability condition for the pressure used. Varying the time creates different heating rates. Ramping simulations use a 4 × 4 × 4 fully occupied methane hydrate unit cell for comparison. All of the temperature ramping cases are conducted at a constant pressure of 50 bar and increasing temperature with 7 different heating rates, shown in Table 2.

**Table 2:** Temperature Ramping Simulation Conditions

simulated time (ns)	pressure (bar)	initial temp. (K)	final temp. (K)	heat rate (TK/s)
1.0	50	100	500	0.4
0.5	50	100	500	0.8
0.4	50	100	500	1.0
0.3	50	100	500	1.3
0.2	50	100	500	2.0
0.1	50	100	500	4.0
0.01	50	100	500	40

A step temperature simulation raises the system temperature instantaneously from a given initial temperature to a final temperature. In this case, the size of the temperature step varies to create different thermal shocks to the system. A 2 × 2 × 2 hydrate system is used to simulate 5 temperature step sizes of Δ*T*, 80, 85, 90, 95, and 100 K above hydrate equilibrium stability conditions 270 K and 50 bar. Simulation conditions for step simulations are shown in Table 3.

**Table 3:** Temperature Step Simulation Conditions

simulated time (ns)	pressure (bar)	initial temp. (K)	final temp. (K)	Δ*T* (K)
5.0	50	270	350	80
5.0	50	270	355	85
5.0	50	270	360	90
5.0	50	270	365	95
5.0	50	270	370	100

### Analysis Methods

There are different methods and criteria to identify and determine the dissociation temperature. This work uses potential energy, mean square displacement (MSD), and change in simulation length (Lx) to analyze the dissociation process from different perspectives. This section aims to downselect the most appropriate properties for this research among the three. The preliminary cases chosen are the simulation results of the ramping simulations at heating rates of 0.4, 0.8, and 4.0 TK/s because the rates are at reasonably distinguishable factors from each other and also because temperature ramping uses 4 × 4 × 4 for a more detailed view of dynamic changes for observation. Further structure analysis for full hydrate systems (to be discussed in the Results section) includes the radial distribution function (RDF), MSD, and analysis of the trajectory files obtained during the simulations.

### Determining Dissociation

**Figure 4:**
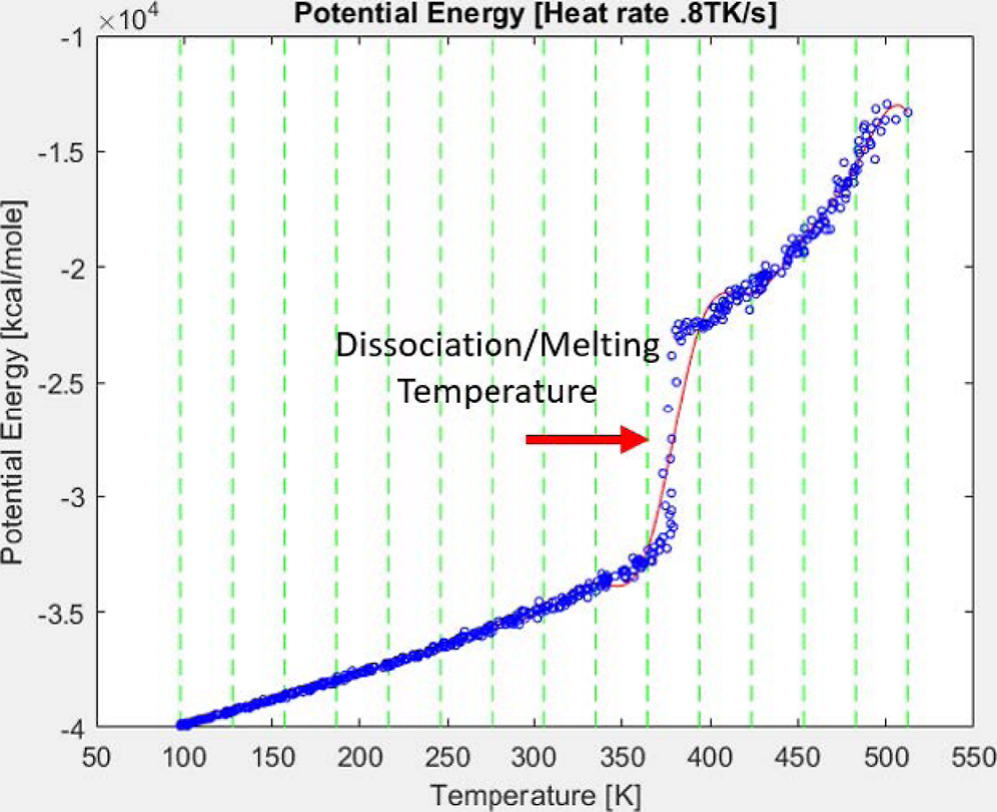
Potential energy–temperature plot at a heating rate of 0.8 TK/s, showing spline approximation.

The potential energy of the system was analyzed to determine the dissociation temperature regime. The principle lies in the change of the internal energy during the phase transition. The internal energy of the system is associated with the energy behind the random movement of the molecules. During phase change, while the temperature of the system, which is defined as the average kinetic energy of all particles of the system, remains statistically constant, a change in the potential energy is observed. With this principle, the approximate dissociation temperature can be observed in a potential energy–temperature plot, as shown in Figure 4, to be the region where the temperature is slowly increasing, while the potential energy increases more dramatically.

To avoid interpretive bias, the dissociation temperature identified in this work was obtained by fitting a spline curve and calculating the inflection point of the curve from its second derivative using MATLAB.^28^ It is clear that the temperature band over which the potential energy changes is quite narrow (less than 10 K).

The MSD of the oxygen molecules can also be used to determine hydrate dissociation. This is because when the dissociation occurs, water molecules, which can be represented by their oxygen atoms for simplicity, break out of the cage and increase significantly their diffusive behavior. A dramatic increase in the MSD values was then observed. The dissociation temperature using this analysis approach was calculated using the same method as the potential energy by fitting a spline curve and calculating the inflection point from its second derivative using MATLAB.

Another approach analyzed in this study was the change in the volume of the simulation box. Before a hydrate dissociates, the volume is constant. When the dissociation process initiates, water cages disintegrate (melt) and methane gas escapes, which leads to a rapid increase in the volume. This principle can therefore be used to calculate methane hydrate dissociation by analyzing the changes in the simulation box length throughout the simulation.

The results of the comparative analysis methods are listed in Table 4. The highest dissociation temperature for all three heating rates was obtained from the potential energy definition. The results obtained from MSD and the length of the simulation box were not consistently higher or lower; that is, there is not a measurable criterion computed as an overall higher or lower temperature for the three heating rates. A higher dissociation temperature was obtained at a heating rate of 4 TK/s using MSD as compared with the box length method, while it was lower for the other two heating rates.

**Table 4:** Dissociation Temperature [K] Calculated Using Potential Energy, MSD, and Cell Length

heating rate (TK/s)	potential energy method (K)	MSD method (K)	length method (K)
0.4	371.5	354.4	365.4
0.8	279.2	367.9	376.6
4.0	409.6	394.1	391.0

Note that the largest discrepancy between dissociation temperature from the 3 methods occurs at the highest heating rate. This inconsistency means that the system needs an adequate amount of time to allow the hydrates and molecules to react and equilibrate appropriately before the occurrence of dissociation can be identified. In particular, the distance-based measures (box length and MSD) must certainly require enough time to allow the atoms to move a noticeable distance even if their lattice bonds have been released. Thus, we can conclude that the definition of dissociation at a molecular level is in itself indistinct.

The results analysis following uses only potential energy to determine dissociation temperature because that measure is the most distinctive and least dependent on curve fitting. The potential energy takes into account the pair and bonding energy as well as angle, dihedral, improper long-range, and fixed energy and, thus, the dissociation temperature can be seen as an effect caused by the change in the potential energy of the atom or the whole system. Dissociation is also reflected fundamentally in the discontinuous jump in potential energy during phase change (moving from one state of matter to another).

## Results and Discussion

### Temperature Step Simulations

For temperature step simulations, the effect of constant step increments of temperature for observing the dissociation process was conducted. The dissociation initiation time at each temperature step was calculated. Similarly, to the previous section (temperature ramping), the results of MSD and (RDF) during the dissociation period are presented.

### Dissociation Starting Time at Different Temperature Steps

The dissociation time is shown in Table 5, which represents the time for the system to start dissociating using both the potential energy and trajectory movements to determine the dissociation initiation. The results show that the system starts to dissociate sooner at a larger Δ*T*, while the system does not dissociate at a Δ*T* of 80 K, which represents the limit of the simulations.

**Table 5:** Time the Hydrate Started to Dissociate at Different Temperature Steps

final temp. (K)	diss. start time (ps)	temp. step size (K)
350	no diss	80
355	265.4	85
360	277.4	90
365	127.4	95
370	57.6	100

**Figure 5:**
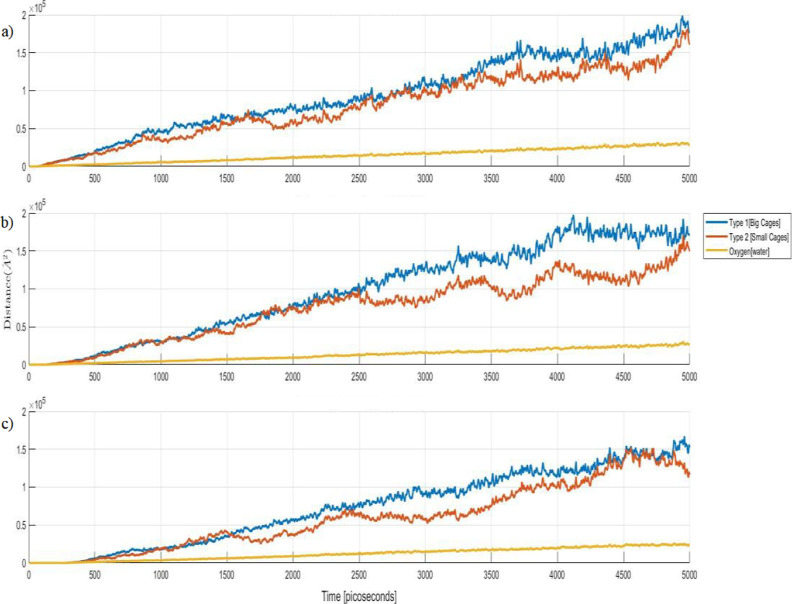
MSD of oxygen atoms representing water molecules in yellow and methane molecules in large cages and small cages in blue and red, respectively, at 370, (a), 365 (b), and 360 K (c).

The plots of the MSD for oxygen and methane in small and large cages during temperature step simulations are shown in Figure 5. The results are very similar for all the 3 selected temperatures, 360, 365, and 370 K, showing a clear difference for oxygen, representing water in solid and later in liquid state, and methane. The methane gas showed more diffusive activity than water.

The diffusion coefficients, as calculated according to Einstein’s relation at constant temperature during the simulated 5 ns for oxygen and methane in small and large cages, are shown in Table 6. While the oxygen diffusion coefficient is clearly lower than the methane diffusion coefficient hosted in both cages, the difference in the diffusion coefficient does not change significantly with the size of the cage occupied. The results are consistent with the MSD analysis.

**Table 6:** Diffusion Coefficient of Oxygen, Methane Type 1, Located in Large Cages, and Methane Type 2, Located in Small Cages

H_2_O		CH_4_	
temperature (*T*)	oxygen	type 1	type 2
370	1.01 × 10^–4^	5.88 × 10^–4^	5.01 × 10^–4^
365	9.22 × 10^–5^	6.77 × 10^–4^	4.62 × 10^–4^
360	8.89 × 10^–5^	5.51 × 10^–4^	4.79 × 10^–4^

**Figure 6:**
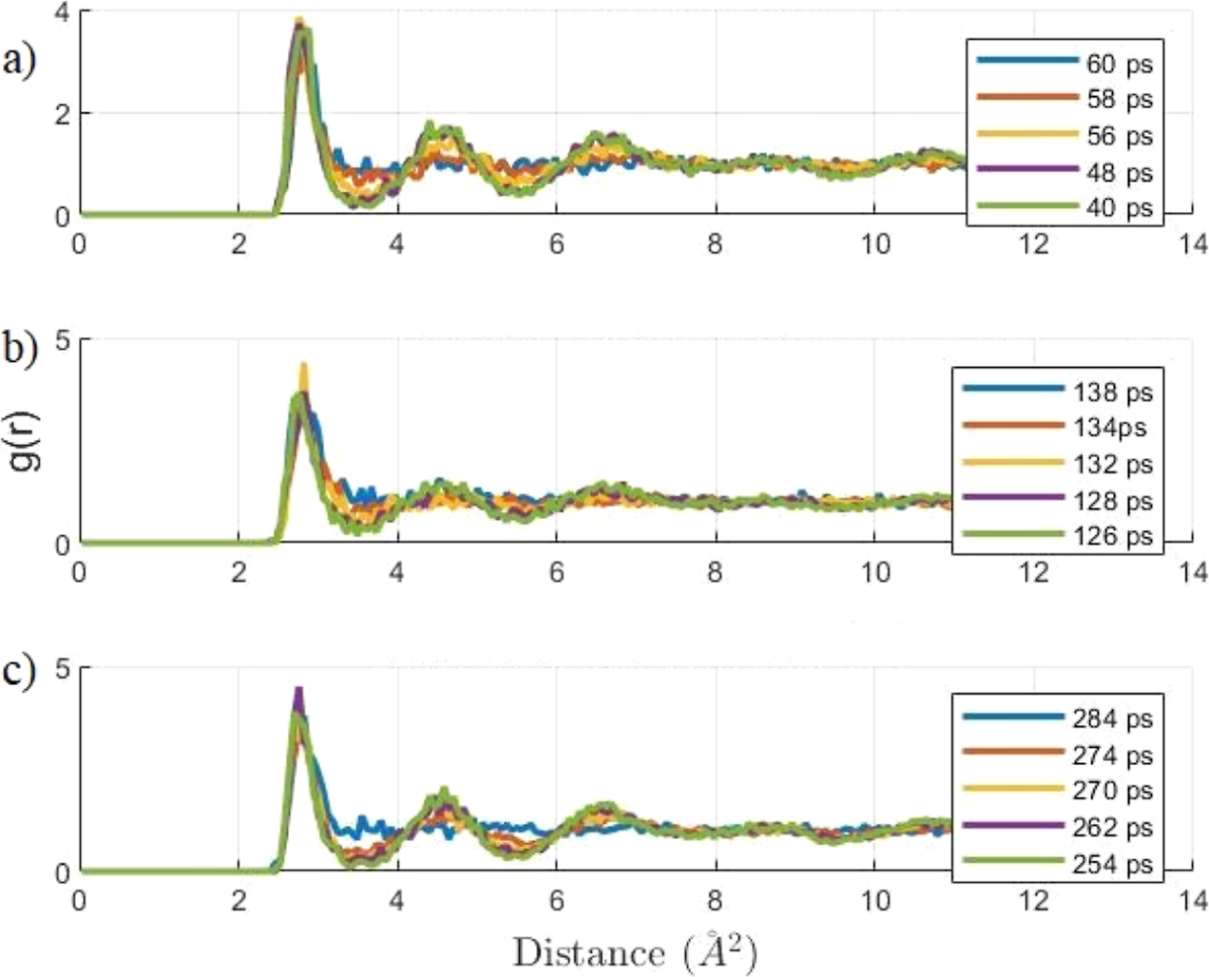
RDF of oxygen–oxygen interactions, representing the structure of water molecules for a temperature step of 370 (a), 365 (b), and 360 K (c) at five different time stages of dissociation.

Like the previous temperature ramping simulations, the RDFs at 5 different time stages of dissociation highlight the changes in the structure. The RDFs at 370, 365, and 360 K are shown in Figure 6, and all 3 plots show a gradual dissociation of the hydrate structure. There is an important aspect to note that the duration of the dissociation process at 370 K was 20 ps, at 360 K, dissociation was 30 ps, and at 365 K, the dissociation was faster at 12 ps. These dissociation time results are distinctly related to the temperature step, but they are approximate time data since the values are limited by the time interval between the frames of the trajectory files.

### Temperature Ramping Simulations

For temperature ramping simulations, the effect of increasing the temperature at different heating rates was analyzed, and the dissociation temperatures at these rates were calculated. The diffusive behavior of methane molecules in large and small cages as well as oxygen atoms, which represent water molecules, were quantified with the MSD. Changes in the hydrate structure were recorded using the RDF of oxygen interactions. Finally, methane molecular trajectories of displacement during dissociation were also analyzed.

### Dissociation Temperature at Different Heating Rates

The results in Table 7 show that the system dissociated at a higher temperature at a higher heating rate. As the temperature increases faster at higher heating rates, hydrate molecules have less response time for the same temperature increase. In other words, the hydrate molecules respond to a higher temperature increase per unit time, and because of this, the dissociation occurs at a higher temperature than if the molecules were given more time to adjust for a slower heating rate.

**Table 7:** Dissociation Temperature of the Methane Hydrate System at Different Heating Rates

heating rate (TK/s)	dissociation temperature (K)
0.4	371.5
0.8	379.2
1.0	387.6
1.3	393.7
2.0	384.3
4.0	409.6
40	436.9

The stability of molecules in hydrate cages throughout the simulation is analyzed using MSDs of oxygen atoms, representing water molecules and methane in large and small cages. In order to observe a clear difference in the MSD results, there are two heating rates selected from Table 7. One is a relatively low heating rate at 0.4 TK/s, and the other is a high heating rate at 4 TK/s.

**Figure 7:**
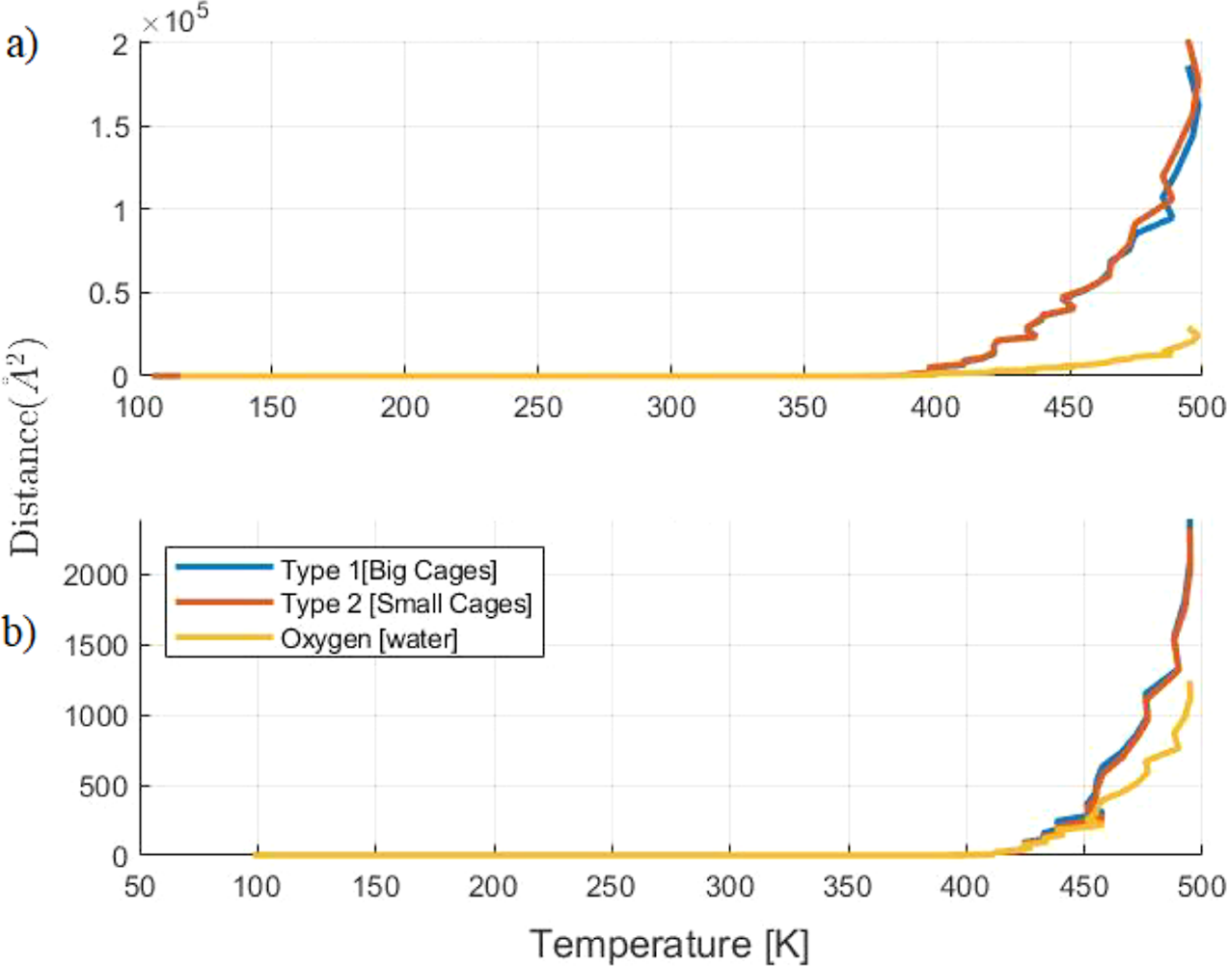
MSD of oxygen atoms representing water molecules in yellow and methane molecules in large cages and small cages in blue and red, respectively. Two heating rates, 0.4 (a) and 4 TK/s (b), are shown.

The results show the distinct difference in dissociation temperature for the system described above, while oxygen atoms exhibit correspondence with an MSD value similar to that of methane at 4 TK/s, as shown in Figure 7. This indicates a similar diffusive behavior for methane and water, particularly at a high heating rate. At a lower heating rate, the MSD of methane in both large and small cages is clearly higher than that of oxygen, which indicates a higher diffusive behavior with respect to water.

### Radial Distribution Function

**Figure 8:**
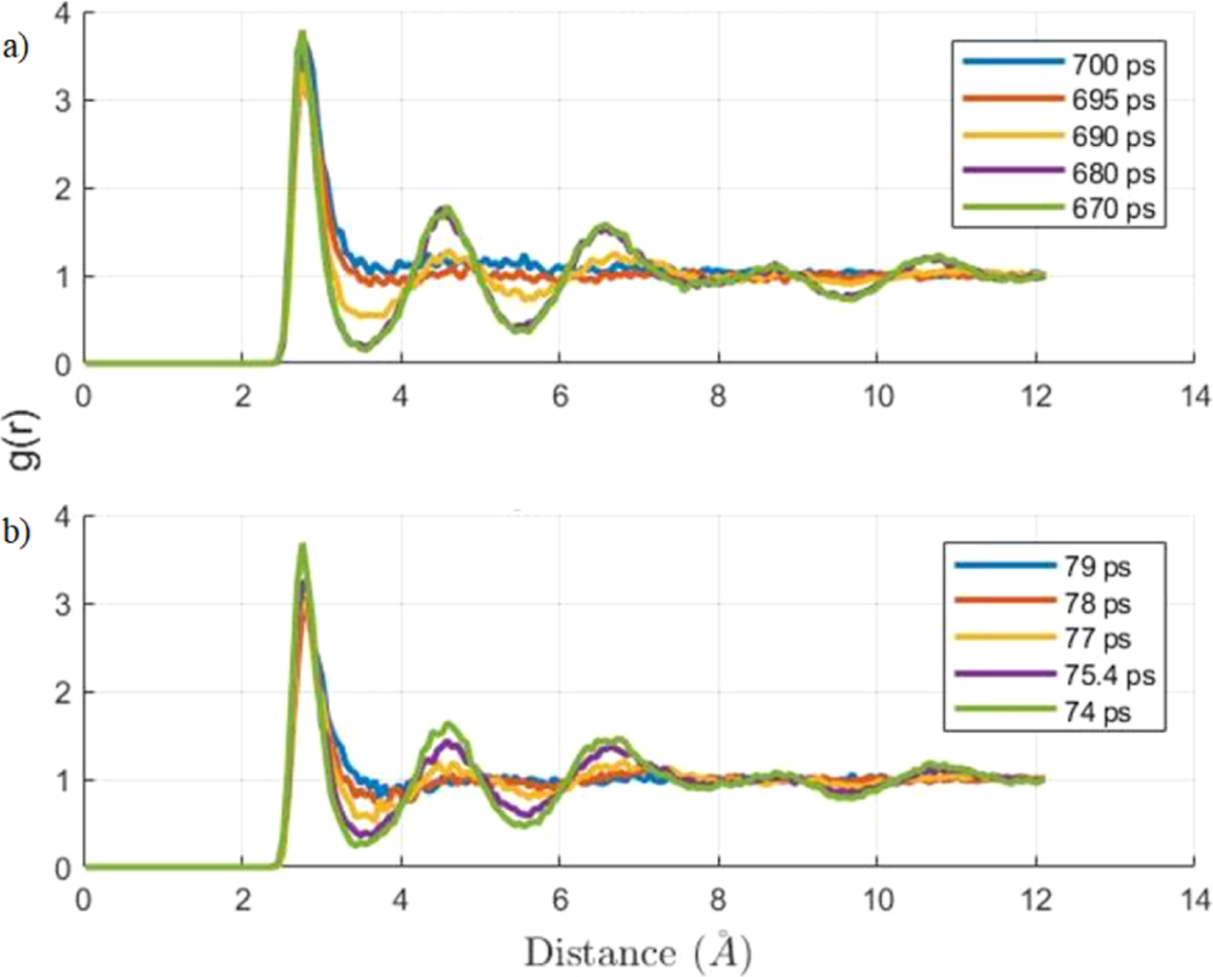
RDF of oxygen–oxygen interaction representation at 5 different time stages of dissociation at a heating rate of 0.4 (a) and 4 TK/s (b).

The RDF information on the evolution in the methane hydrate structure during dissociation is plotted in Figure 8. The RDF of oxygen–oxygen interaction representing the water molecules and the hydrate cages formed by them, which has a cutoff distance of 12 Å, the length of the unit cell, at 5 different stages of dissociation determined by potential energy plots and trajectory files, was extracted. To study the influence of the heating rate, as in the MSD study, the results of the RDFs at a heating rate at 0.4 and 4 TKs were examined. The results in Figure 7 clearly show characteristics of a solid methane hydrate at an early time (e.g., 670 ps), with distinct valleys representing an ordered methane hydrate crystalline structure. The structure gradually disappears at a later time until the RDF curve flattens, as is characteristic of liquid water. The comparison of heating rate shows that more time is needed for a methane hydrate to dissociate at a heating rate of 0.4TK/s as compared to a duration of 5 ps at a high heating rate of 4 TK/s, which represents a low heating rate with a duration of 30 ps.

### Homogeneous Dissociation

**Figure 9:**
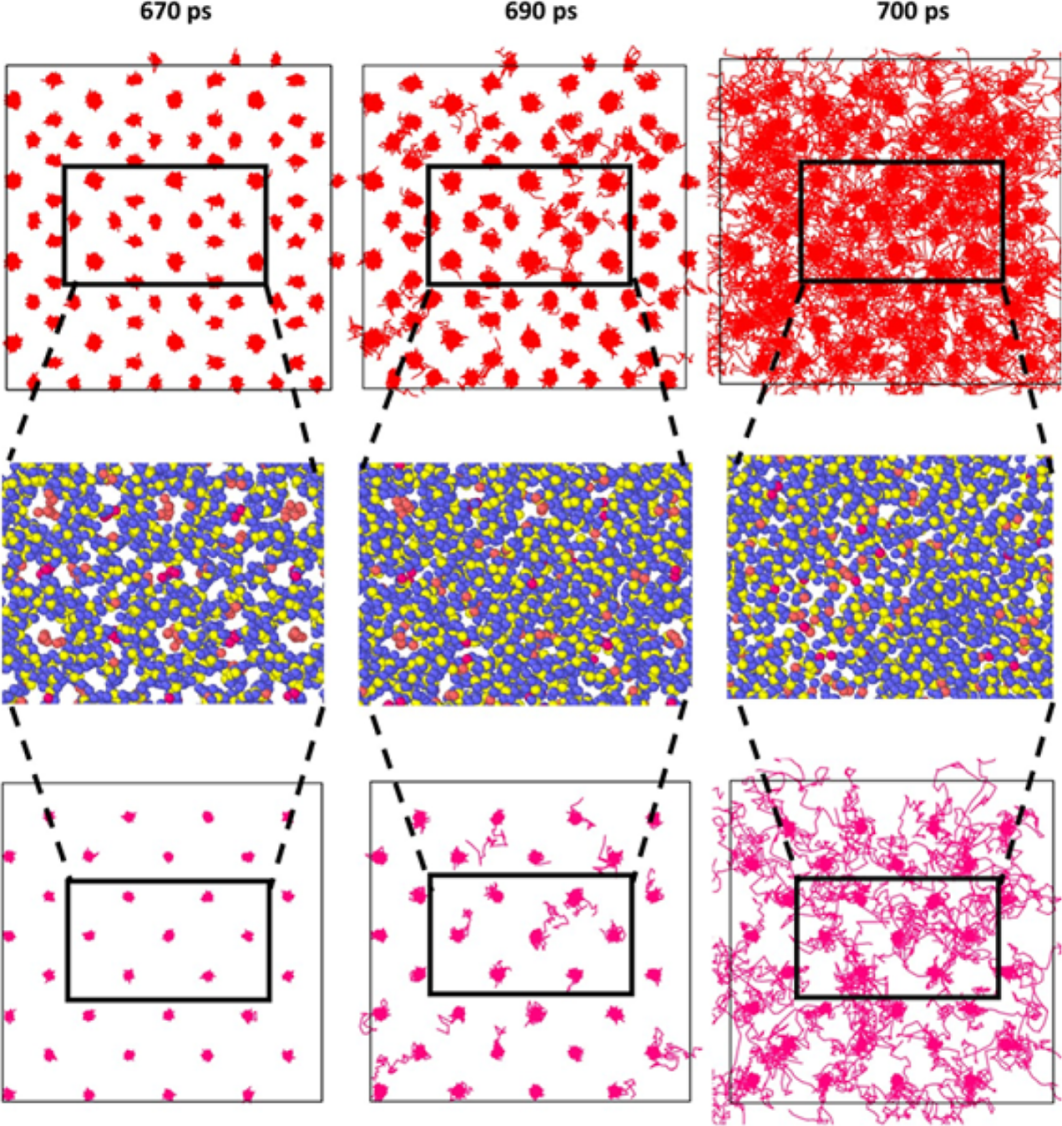
Methane molecule displacement located at large cages (top) and small cages (bottom) and as an enlarged portion of the hydrate structure (middle) are shown at a heating rate of 0.4 TK/s at different times of dissociation in picoseconds.

**Figure 10:**
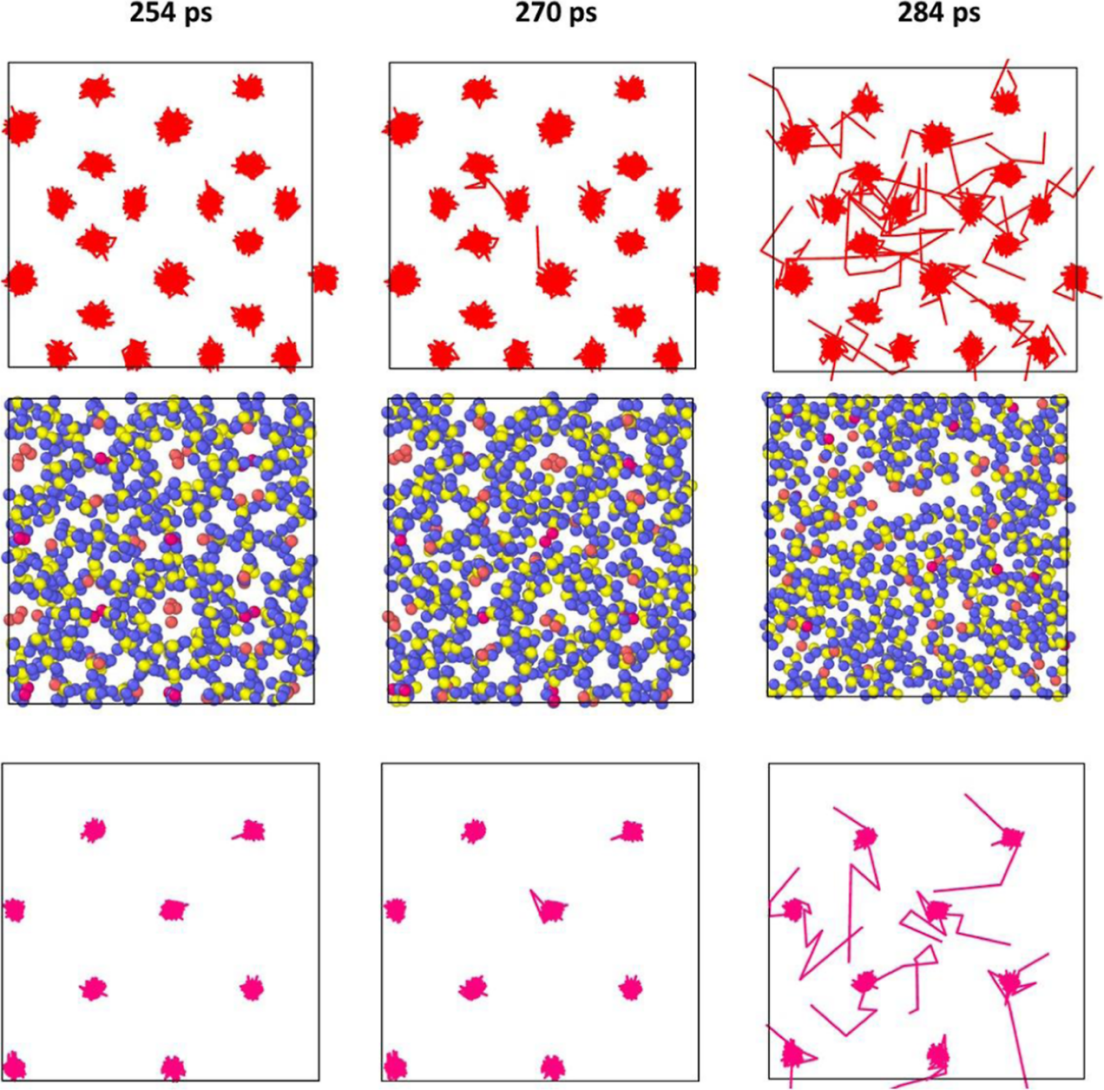
Methane molecule displacement located at large cages (top) and small cages (bottom) at a constant temperature of 360 K at different times of dissociation in picoseconds.

The difference in the MSD of individual methane molecules in small and large cages in all simulations was not significant enough to represent a heterogeneous dissociation. This observation was supported by the analysis of individual and systemwide molecular displacements. The collective displacements throughout three different times of dissociation for both ramping and step simulations are shown in Figures 9 and 10. Visually, there is no clear “starting point” for dissociation.

**Figure 11:**
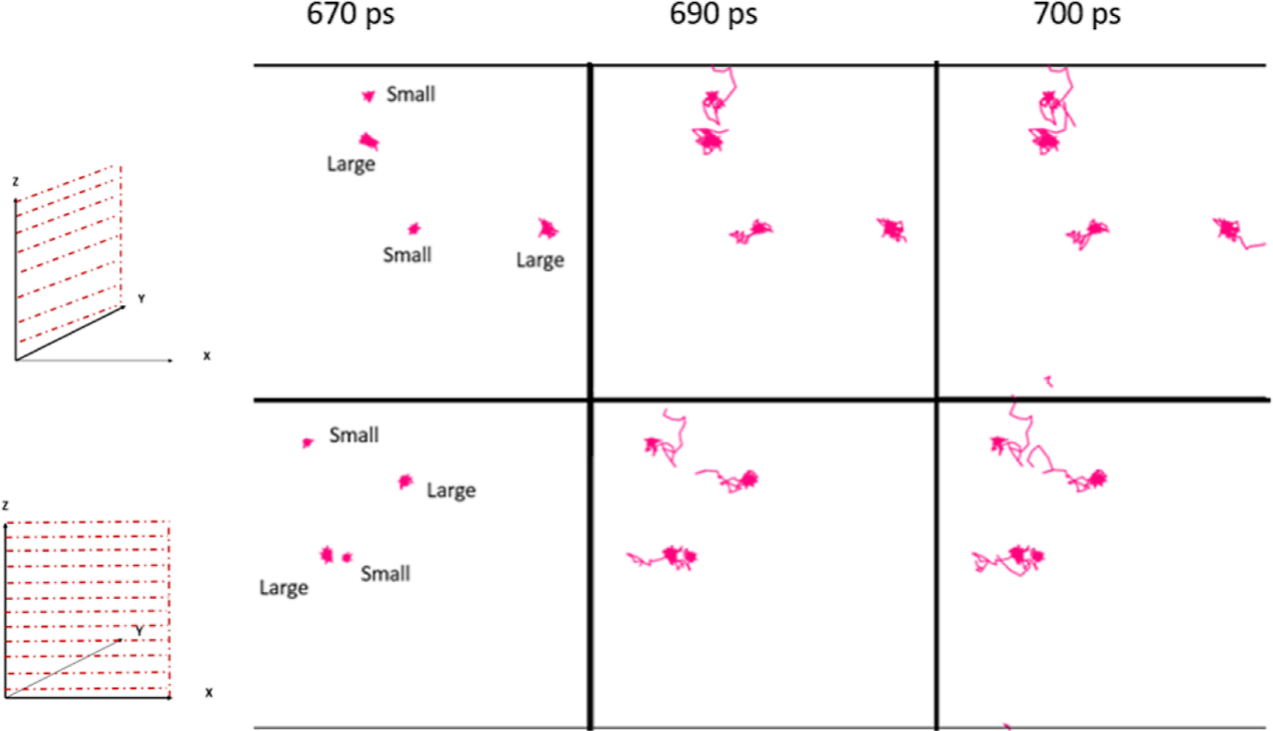
Individual displacements of methane contained in small and large cages from views at two different planes *YZ* plane (top) and *XZ* plane (bottom) at three different times during dissociation with a heating rate of 0.4 TK/s.

For temperature ramping simulations, the individual displacements of methane in two locations in small and large cages were analyzed and are shown in Figure 11. Individual displacements on planes *YZ* and *XZ* of the same methane molecules were analyzed to determine whether displacements were similar for the molecules, independent of the plane.

Both MSD and RDF analyses support the hypothesis that homogeneous dissociation occurs at various temperature steps.

### Hydrate Structure I Coordination Analysis

**Figure 12:**
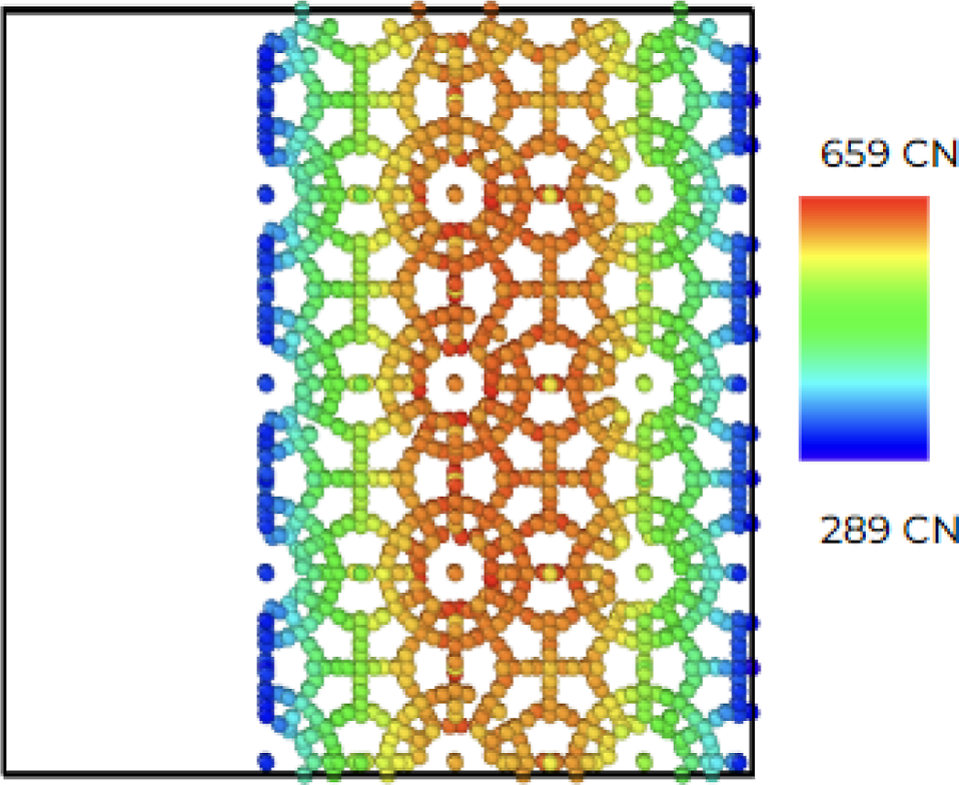
Coordination analysis in the (0 1 0) plane 3 × 4 × 4 hydrate supercell.

**Figure 13:**
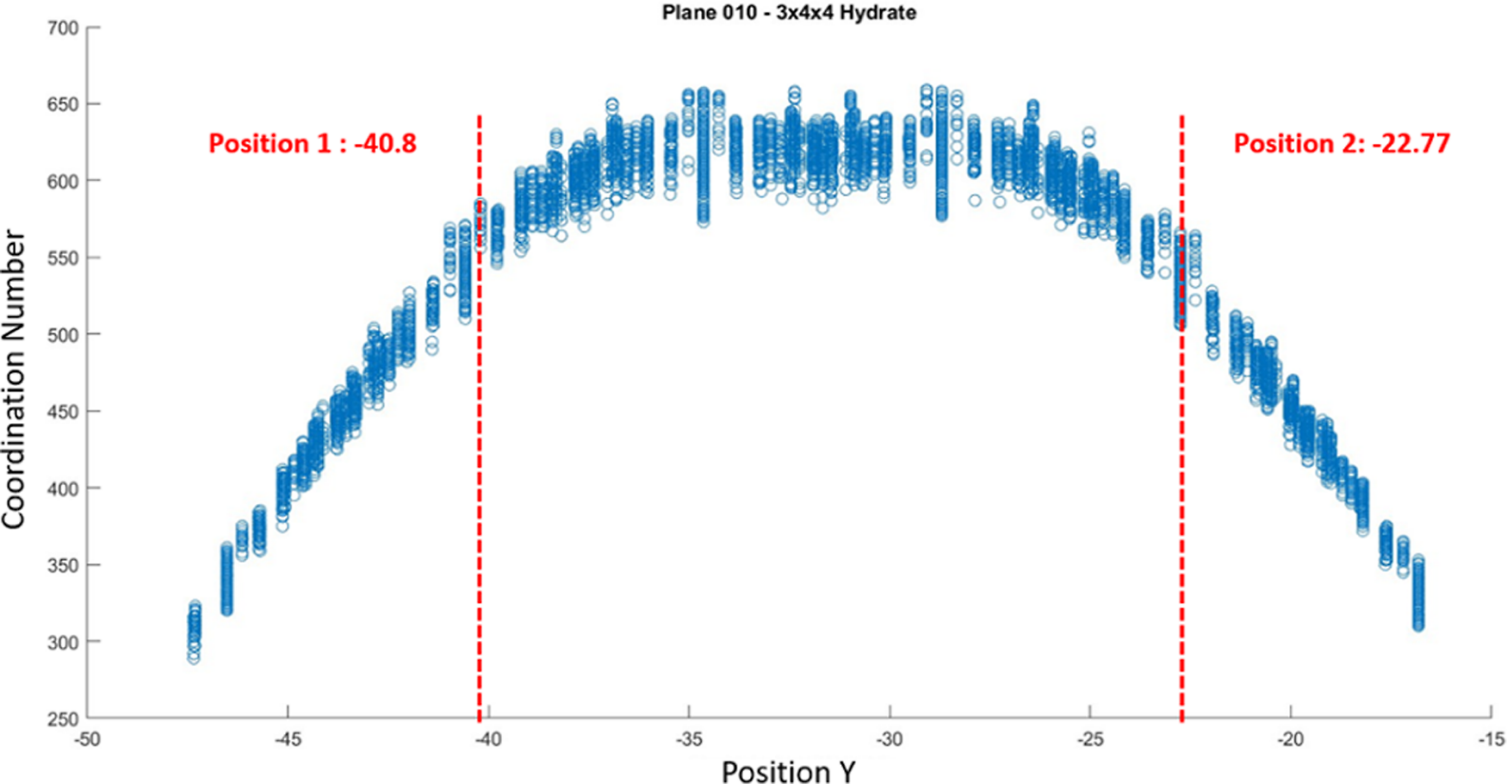
Position of the free surface in the *Y* axis.

The methane hydrate dissociation behavior from a molecular dynamics perspective and using LAMMPS from various aspects are described above, and these systems are studied assuming infinite boundaries. The next step to observe a more realistic exchange is to focus near the hydrate surface. Therefore, we explored the concept of a hydrate-free surface at the structural level to differentiate it from the bulk behavior. A coordination analysis was performed in a sliced hydrate system (3 × 4 × 4) creating two free surfaces along the *Y* axis. Figure 12 shows the hydrate cell in a 3 × 4 × 4 structure, and the coordination number (CN) is based on the RDF data. The CN, also called the hydration number, is plotted with a scaled colorbar from blue to red (small to large). The results show that the coordination number decreases as it approaches the free surface and becomes uniform in the middle. Figure 13 shows the plot of the coordination number to the exact position on the *Y* axis, and it clearly shows that the free surface boundaries are located at coordinates *Y*: −40.8 and *Y*: 22.77 along the hydrate.

## Conclusions

This work presents the molecular dynamic scope of the process for the temperature step and ramping for methane hydrate to induce dissociation. Methane molecules in large and small cages were classified and analyzed to observe and monitor changes in large or small cages for dissociation. Both types of simulations showed homogeneous dissociation, meaning that there was no significant difference in the time of dissociation between small and large cages. This was determined by the diffusion coefficient in the temperature step simulations and the difference in MSD in ramping simulations, and molecular displacements for both were also analyzed.

In temperature step simulations, dissociation occurred earlier and in less time at a larger Δ*T*, while cases with a temperature step under 355 K did not dissociate during the simulated 5 ns. The difference between the MSDs of methane molecules in small and large cages increased significantly at the end of the dissociation time. The behavior of the MSD after dissociation, which was calculated with the potential energy and RDFs, can be described as random. The diffusion coefficient for methane molecules in large cages was slightly higher than that for the methane molecules trapped in small cages.

Ramping simulations showed a relatively large uncertainty for dissociation temperature calculations; this can be caused by the fast increase in temperature without letting the system equilibrate; slower heating rates showed a more defined potential energy and MSD curve, which are better for the calculation of thermodynamic properties.

To conclude, for dissociation analysis, temperature step simulations have the benefits of less computational time for reaching the same homogeneous dissociation at a reasonable temperature as ramping, which is better for analyzing the relationship between physical and thermodynamic properties. Slow heating rates of the temperature ramping show a slower structure change, which can allow the analysis of specific cage dissociation more thoroughly.

The effects of pressure, temperature, and cage occupancy for methane have been widely studied at different scales, including at the molecular scale, as in this work. Moreover, most molecular dynamics studies were accomplished by simulating a sole methane hydrate system, meaning merely one hydrate cell and only a single guest molecule type.

The coordination analysis was also conducted for the free surface as the step to prepare and understand CO_2_ sequestration and the different structural concepts of the free surface and bulk.

Further experimental and theoretical studies are needed to optimize and understand the exchange process with carbon dioxide.
